# Measuring Vitamin Literacy and Information-Seeking Behavior

**DOI:** 10.7759/cureus.60704

**Published:** 2024-05-20

**Authors:** Anas Alhur, Afrah A Alhur, Suha Alqahtani, Hajar Al Obaid, Rahaf Mohammed, Ibrahim Al-Humam, Saeed Buhayr, Refal Altowairqi, Wajan Al-Shahrani, Abdullah Alshadidi, Husam Alghamdi, Sondos Adel Qasim, Waiel A Alqabail, Wala H Albanai, Fatimah O Alanezi

**Affiliations:** 1 Health Informatics, University of Hail College of Public Health and Health Informatics, Haʼil, SAU; 2 Nutrition, University of Hail, Haʼil, SAU; 3 College of Pharmacy, King Khalid University, Abha, SAU; 4 College of Pharmacy, University of Tabuk, Tabuk, SAU; 5 Pharmacy, Al-Jaber Eye and ENT hospital, Al Hofuf, SAU; 6 College of Pharmacy, Taif University, Taif, SAU; 7 Department of Nursing, Qatif Central Hospital, Dhahran, SAU; 8 Second Cluster Al Yamamah Hospital, Ministry of Health, Riyadh, SAU

**Keywords:** nutrition education, public health, supplement use, dietary decisions, information-seeking behaviors, vitamin literacy

## Abstract

Background

The significance of vitamins in maintaining health necessitates a comprehensive understanding among the population, which is critical for making informed decisions pertaining to diet and vitamin supplementation. Nevertheless, there is a notable deficiency in research regarding the mechanisms through which individuals in the Kingdom of Saudi Arabia (KSA) acquire and utilize vitamin-related knowledge, marking a considerable void in public health and nutrition education.

Methods

This study proposes a quantitative, cross-sectional analysis to evaluate vitamin literacy and information-seeking behaviors within KSA’s diverse demographic sectors. Survey instruments will be utilized to ascertain the primary sources from which individuals derive vitamin information and to analyze the factors that influence the integration of this knowledge into daily health practices.

Results

Analysis showed significant variance in vitamin literacy with age and education acting as key differentiators. Kruskal-Wallis tests indicated significant differences in self-rated vitamin knowledge across age groups (p < 0.001), and Chi-square tests confirmed the association between education level and supplement use (p = 0.0022). The majority of participants demonstrated moderate knowledge of vitamins, yet a discrepancy exists between this knowledge and dietary application. Trust in information sources emerged as a significant predictor of dietary change (Chi-square, p < 0.001), although a substantial portion of participants did not actively incorporate vitamin knowledge into their daily diet.

Conclusion

The enhancement of vitamin literacy is paramount for the formulation of effective public health strategies. Our findings suggest that targeted educational programs, especially for younger and less-educated populations, are crucial to bridge the gap between vitamin knowledge and its application in daily diet. In addition, efforts should focus on improving the credibility of information sources, as this significantly influences dietary changes. These initiatives can lead to more informed decision-making regarding diet and vitamin supplementation, ultimately fostering healthier living practices and reducing the occurrences of vitamin-related health issues within the KSA populace. This research contributes substantively to the development of tailored educational initiatives and informed policymaking, guiding future efforts to advance nutrition guidelines and public health in the region.

## Introduction

Understanding how vitamins impact health and making informed decisions about their use is crucial for public health. Vitamin literacy refers to an individual's knowledge and understanding of vitamins, including their functions, sources, recommended daily intake, and potential health implications of deficiencies or excess intake. This comprehensive grasp is essential for promoting health and preventing diseases through appropriate dietary choices.

Studies indicate that while people recognize vitamins are beneficial, they often lack detailed knowledge about their specific roles and recommended amounts. For instance, in the UK, awareness of the importance of vitamin D is common, especially in winter or when sun exposure is minimal. However, deeper knowledge of vitamin D's biological roles and dietary sources is often superficial [1].

Information-seeking behavior is how individuals actively search for, evaluate, and use information to meet their needs and make health-related decisions. This behavior is critical in how people gather and apply vitamin knowledge to their dietary practices. A diet and nutrition study revealed that even though online health websites are popular for information, individuals trust healthcare professionals and scholarly articles more. This highlights a gap between the popularity of information channels and their credibility, with the latter having more influence on dietary habits [2]. Language and cultural differences also play a role in information-seeking, as seen in a study among Palestinians where distrust in online sources and a lack of Arabic resources were significant obstacles [3].

In Saudi Arabia, the situation is unique due to sociodemographic factors, cultural practices, and resource availability that shape how vitamin knowledge is used. Preliminary data suggest that there is a significant lack of vitamin literacy, particularly regarding the roles of vitamins in preventing chronic diseases [4]. This deficiency is evident in dietary patterns that often do not align with nutritional recommendations, leading to issues such as vitamin D deficiency despite ample sunlight throughout the year.

Multiple factors, including sociodemographic characteristics, cultural practices, and resource availability, affect how vitamin knowledge is utilized. Research in Ethiopia indicated that farmers' views and actions on malnutrition and nutrient deficiencies are influenced by demographic factors, particularly gender differences in household nutrition choices [5]. In China, lifestyle elements like diet, exercise, and sun protection were connected to vitamin D levels, showing the importance of lifestyle in using vitamin knowledge [6]. In addition, the use of vitamin A supplements among Ethiopian children was closely linked to maternal knowledge, emphasizing how maternal education affects the practical use of vitamin knowledge [7].

Taken together, these studies show that while having knowledge about vitamins is essential, the use of this knowledge in practice is influenced by factors, such as trust, accessibility, economic conditions, and cultural values. Understanding these aspects is key to devising successful methods to enhance vitamin knowledge and its practical use among different communities, especially in Saudi Arabia.

The primary objective of this research is to assess vitamin knowledge in Saudi Arabia, pinpoint the key sources of vitamin information, and analyze the factors influencing the application of this knowledge in everyday dietary and health choices. This comprehensive approach is designed to deepen our understanding of how to encourage healthy eating and supplement use based on informed decisions. The public health implications of our findings indicate that targeted educational initiatives and policy interventions are essential to enhance vitamin literacy and empower individuals to make well-informed decisions regarding their health and diet.

## Materials and methods

Research design

This study employed a quantitative, cross-sectional design to assess vitamin literacy across diverse groups in Saudi Arabia. By collecting data at a single point in time, this method facilitated insights into the current knowledge and behaviors related to vitamin intake. The cross-sectional approach was instrumental in identifying general patterns and relationships within the data, which are crucial for developing public health strategies.

Data collection

Data were gathered using a comprehensive online questionnaire designed to explore various aspects of vitamin literacy. The questionnaire covered demographic details, knowledge about vitamins, methods of seeking health information, and the application of this knowledge in daily diets, with a focus on individuals with diabetes. The questionnaire featured multiple-choice and Likert scale questions to measure factual knowledge and attitudes. Prior to its official deployment, the questionnaire underwent a rigorous validation process, including the following: 1) face validity: Ensured through expert reviews to confirm that the questions adequately represented the intended content; 2) content validity: established by consulting with nutritionists and public health experts to ensure the questions accurately captured the full range of vitamin literacy concepts. This process ensured both the validity and reliability of the instrument.

The survey was conducted on a secure online platform, adhering to international data protection standards to maintain participant anonymity and confidentiality. Recruitment targeted a diverse demographic widely represented on social media platforms like WhatsApp, Twitter, and Snapchat, aiming to include a broad range of ages, educational backgrounds, and urban and rural residents of Saudi Arabia.

Sampling

The study utilized stratified random sampling to accurately reflect the population of Saudi Arabia. This method accounted for geographic, socioeconomic, and cultural differences to collect a representative set of responses. Sample size determination was based on a power analysis to achieve a 95% confidence level and a 5% margin of error, ensuring the statistical significance of the study findings.

Data analysis

Data from the questionnaire were analyzed using IBM SPSS Statistics for Windows, version 26.0 (released 2019, IBM Corp., Armonk, NY). The analysis incorporated descriptive statistics to summarize the data and inferential statistics to identify relationships and trends. Specifically, descriptive statistics were used to summarize the demographic information and the distribution of responses, while inferential statistics, including regression analysis and ANOVA, were applied to explore how vitamin literacy impacts dietary choices, particularly among individuals with diabetes. In addition, Kruskal-Wallis tests and Chi-square tests were utilized to assess differences and associations among various demographic groups.

Ethical considerations

The research followed strict ethical guidelines approved by the University of Hail’s Research Ethics Committee, under the identifier H-2024-039. Informed consent was obtained from all participants, who were informed about the study's purpose and their right to withdraw at any time. Measures were taken to ensure the privacy and confidentiality of all participants, demonstrating the study's commitment to ethical research practices.

## Results

In the demographic analysis of the study participants, notable trends emerged across gender, age, and education levels. The population comprised 1,928 females (65.10%) and 1,033 males (34.90%). Age-wise, the groups of 18-25 and 26-35 were most represented with 793 (26.80%) and 750 (25.30%) individuals, respectively, followed by the 36-45 age group with 500 individuals (16.90%), the 46-55 group with 175 (5.90%), and the over-55 group with 35 (1.20%). Regarding education, 2,066 participants (69.80%) held a Bachelor's degree, 436 (14.70%) were high school graduates, 376 (12.70%) had a graduate or professional degree, and 83 (2.80%) had some high school education, as detailed in Table 1.

**Table 1:** Demographic information

Demographic variable	Category	Frequency	Percent
Gender	Male	1,033	34.90%
	Female	1,928	65.10%
Age group	18-25	793	26.80%
	26-35	750	25.30%
	36-45	500	16.90%
	46-55	175	5.90%
	Over 55	35	1.20%
Education level	Some high school	83	2.80%
	High school graduate	436	14.70%
	Bachelor’s degree	2,066	69.80%
	Graduate or professional degree	376	12.70%

In assessing self-rated knowledge about vitamins and their functions, the participants exhibited a wide range of self-perceptions. A substantial portion of the participants rated their knowledge as "average" with 987 individuals (33.33%) or "very good" with 960 individuals (32.42%). However, there was also a notable representation of individuals who considered their knowledge to be "poor," totaling 786 individuals (26.55%), which highlights a significant gap in vitamin literacy among the population. A smaller group rated their knowledge as "good," comprising 228 individuals (7.7%), indicating modest confidence in their understanding of vitamins and their functions.

Regarding awareness of the consequences of vitamin deficiencies, a majority of the participants (2,311 individuals, 78.05%) acknowledged being aware. By contrast, a smaller segment of the sample, 650 individuals (21.95%), reported a lack of awareness regarding the repercussions of inadequate vitamin intake. This contrast underscores the varying levels of health consciousness and information assimilation within the study cohort. For additional details, refer to Table 2.

**Table 2:** Vitamin literacy

Questionnaire item	Response options	Frequency	Percentage (%)
How would you rate your knowledge of vitamins and their functions?			
	Poor	786	26.55
	Average	987	33.33
	Good	228	7.7
	Very Good	960	32.42
Are you aware of the consequences of vitamin deficiencies?			
	No	650	21.95
	Yes	2311	78.05

Our study showed varied levels of engagement in seeking vitamin-related information among participants. A total of 40.39% (1,196 individuals) reported frequently seeking information about vitamins, while 23.07% (683 individuals) stated they always do. By contrast, 24.38% (722 individuals) occasionally seek such information, and 12.16% (360 individuals) never do.

Concerning the primary sources for vitamin information, books and magazines were the most common, used by 75.97% (1,977 individuals). The Internet was the next most utilized source at 29.14% (758 individuals), followed by consultations with health professionals at 8.69% (226 individuals).

In terms of confidence in the accuracy of the information found, 68.97% (1,794 individuals) were somewhat confident, while 14.30% (372 individuals) were not confident about the reliability of the vitamin information they accessed, as seen in Table 3.

**Table 3:** Information seeking behavior

Questionnaire item	Response options	Frequency	Percentage (%)
How often do you actively seek out information regarding vitamins?	Never	360	12.16
	Occasionally	722	24.38
	Frequently	1,196	40.39
	Always	683	23.07
Where do you primarily seek information about vitamins?	Health professionals	226	8.69
	Internet	758	29.14
	Books/magazines	1,977	75.97
How confident are you in the accuracy of the vitamin information you find?	Not confident	372	14.3
	Somewhat confident	1,794	68.97

When assessing whether participants considered their vitamin needs when making dietary choices, responses varied, reflecting a diverse approach to nutrition. A notable 40.59% (1,202 individuals) of respondents always take their vitamin needs into account when choosing foods, suggesting a proactive stance toward nutrition. Conversely, 14.42% (427 individuals) never consider vitamin needs, which may point to a potential area for public health intervention. Some individuals do so rarely (4.05%, 120 individuals) or sometimes (26.98%, 799 individuals), while 13.95% (413 individuals) often consider their vitamin needs.

The participants were also asked if their current diet reflects their understanding of vitamin needs. A total of 37.39% (1,107 individuals) feel that their diet partially reflects their vitamin knowledge, whereas 19.28% (571 individuals) believe it completely does. However, 35.43% (1,049 individuals) feel their diet does not reflect their vitamin knowledge at all, highlighting a discrepancy between knowledge and practice. A smaller group feels their diet only slightly aligns with their knowledge (7.9%, 234 individuals).

Furthermore, the likelihood of changing one's diet in response to new vitamin-related information was explored. A significant majority (59.41%, 1,759 individuals) stated that they are likely to change their diet based on new information. While 25.06% (742 individuals) are unsure, a smaller fraction is very likely (2.5%, 74 individuals) or have already changed their diet (4.63%, 137 individuals). However, some remain unlikely to make changes (8.21%, 243 individuals), and a few are very unlikely to do so (0.2%, six individuals), as demonstrated in Table 4.

**Table 4:** Factors influencing vitamin knowledge application

Questionnaire item	Response options	Frequency	Percentage (%)
Do you consider your vitamin needs when making dietary choices?			
	Never	427	14.42
	Rarely	120	4.05
	Sometimes	799	26.98
	Often	413	13.95
	Always	1202	40.59
Does your current diet reflect your understanding of your vitamin needs?			
	Not at all	1049	35.43
	Slightly	234	7.9
	Partially	1107	37.39
	Completely	571	19.28
How likely are you to change your diet based on new vitamin-related information?			
	Very unlikely	6	0.2
	Unlikely	243	8.21
	Unsure	742	25.06
	Likely	1759	59.41
	Very likely	74	2.5
	Already changed	137	4.63

Regarding vitamin supplement intake, the participants reported varying frequencies, with an average score of 1.615 and a standard deviation of 0.961. This variation points to a diversity of attitudes toward supplementation. A significant 42.55% (1,260 individuals) of the study population takes vitamin supplements occasionally, while 17.66% (523 individuals) do so regularly, reflecting a proactive approach to supplementing diet with additional nutrients. Conversely, 16.51% (489 individuals) have never taken supplements, and 23.24% (688 individuals) have considered it but have not taken the step, highlighting potential areas for public health education and intervention.

When deciding which vitamin supplements to take, the majority of supplement users rely on personal research (54.64%, 1,618 individuals), indicating a proactive and self-directed approach to health. Others prefer to seek advice from various sources, with 25.97% (769 individuals) basing their decisions on advice from friends, family, or peers and 19.39% (574 individuals) on healthcare professional advice. The mean decision-making score of 0.647 with a standard deviation of 0.785 suggests that while many participants are self-informed, a considerable number still value external guidance.

The likelihood of initiating vitamin supplement use based on new information was also probed, with a mean response score of 1.392 and a standard deviation of 1.334, indicating a moderate openness to change. While a sizable portion of respondents (40.22%, 1,191 individuals) find it very unlikely to start taking supplements based on new information, 20.74% (614 individuals) are likely to consider it. Some respondents are unsure (21.99%, 651 individuals) or unlikely (11.75%, 348 individuals) to start supplementation, and a small fraction is very likely to do so (5.27%, 156 individuals), reflecting a spectrum of openness to adopting new health practices, as indicated in Table 5.

**Table 5:** Vitamin supplementation

Questionnaire item	Response options	Frequency	Percentage (%)
Do you take any vitamin supplements?			
	No, never	489	16.51
	No, but considered	688	23.24
	Yes, occasionally	1260	42.55
	Yes, regularly	523	17.66
If you take supplements, how did you decide which ones to take?			
	Personal research	1618	54.64
	Advice from others	769	25.97
	Healthcare advice	574	19.39
How likely are you to start taking vitamin supplements based on information you've recently learned?			
	Very unlikely	1191	40.22
	Unlikely	348	11.75
	Unsure	651	21.99
	Likely	614	20.74
	Very likely	156	5.27

The comparison of vitamin literacy across different age groups was conducted using a Kruskal-Wallis test, yielding a statistic of 29.33 with a p-value of 0.00001998. This statistical analysis reveals significant differences in self-rated vitamin knowledge among various age groups. The p-value, substantially below the conventional threshold of 0.05, indicates that the differences in vitamin knowledge across age groups are statistically significant. This result shows that vitamin knowledge varies among age groups, which is critical for understanding the distribution of vitamin literacy in the population. For more details, refer to Table 6.

**Table 6:** Comparison of vitamin literacy across age groups Interpretation: Statistically significant differences in self-rated vitamin knowledge across different age groups were observed, suggesting that respondents' age may influence their perceived knowledge about vitamins.

Analysis type	Kruskal-Wallis test
Statistic	29.33
p-value	0.00001998

The influence of education on vitamin supplementation practices was assessed using a Chi-square test, which yielded a statistic of 24.1 and a p-value of 0.0022. This statistical analysis indicates a significant association between the highest level of education completed and the practice of taking vitamin supplements. With a p-value of 0.0022, which is below the conventional significance level of 0.05, we reject the null hypothesis of no association, as shown in Table 7.

**Table 7:** Influence of education on vitamin supplementation practices Interpretation: There is a statistically significant association between a respondent's education level and their vitamin supplementation practices, indicating that education may influence the decision to take vitamin supplements.

Analysis type	Chi-square test
Statistic	24.1
p-value	0.0022

The relationship between confidence in vitamin information and the likelihood of changing diet was examined using a Chi-square test, yielding a statistic of 261.81 and a p-value of 4.56 × 10^-47^. This statistical analysis reveals a highly significant association between individuals' confidence in the accuracy of vitamin information and their propensity to make dietary changes based on new information. With a p-value much smaller than the conventional significance level of 0.05, we reject the null hypothesis of no association, as seen in Table 8.

**Table 8:** Relationship between confidence in vitamin information and likelihood to change diet Interpretation: A statistically significant association was found between individuals' confidence in the accuracy of the vitamin information they find and their likelihood to make dietary changes based on new information, suggesting that the trustworthiness of information sources might influence health-related behaviours.

Analysis type	Chi-square test
Statistic	261.81
p-value	4.56 × 10^-47^

The analysis of vitamin knowledge across different vitamin supplementation groups was conducted using a Kruskal-Wallis test, resulting in a statistic of 183.86 and a p-value of 1.11 × 10^-38^. This statistical analysis indicates significant differences in self-rated vitamin knowledge among the various supplementation groups. With a p-value much smaller than the conventional significance level of 0.05, we reject the null hypothesis of no difference in vitamin knowledge across supplementation groups. For more information, see Table 9 and refer to Appendix A, B, and C for the full questionnaire on vitamin literacy and information-seeking behavior.

**Table 9:** Vitamin knowledge across vitamin supplementation groups Interpretation: Statistically significant differences in self-rated vitamin knowledge across different vitamin supplementation groups were observed, indicating that the decision to take vitamin supplements could be associated with perceived knowledge about vitamins.

Analysis type	Kruskal-Wallis test
Statistic	183.86
p-value	1.11 × 10^-38^

## Discussion

This research provides valuable insights into vitamin awareness and information-seeking behaviors among individuals in Saudi Arabia, with broader implications for public health education and dietary habits. By integrating our findings with existing studies, we emphasize the critical role of education and active information-seeking in enhancing vitamin knowledge.

Our analysis revealed notable differences in vitamin awareness across various ages and educational levels, aligning with previous studies [7]. Younger individuals, particularly those aged 18-25 and 26-35, were more active in seeking information about vitamins, likely due to their regular use of digital media and educational environments that promote health education [8]. Higher education levels were associated with greater understanding and effective use of health information [9], suggesting that integrating comprehensive health and nutrition education into school curricula could encourage healthy behaviors from an early age.

The study highlighted diverse methods of seeking information, with a strong reliance on books and magazines. While the internet was frequently used, its reliability was perceived to be lower than that of health professionals, indicating a significant gap between information availability and trustworthiness [10-13]. This gap underscores the need for trustworthy online health resources, especially for younger individuals who primarily seek information online.

Participants' confidence in the accuracy of the information they found was directly linked to their willingness to change their diets based on that information. Those who trusted their sources were more likely to adjust their diets in response to new vitamin-related knowledge, highlighting the importance of enhancing the credibility of health information sources [14]. Educational initiatives should leverage collaborations with health professionals to provide verified content through engaging channels targeted at young people.

Despite adequate vitamin knowledge among some participants, many did not consistently apply this knowledge when making food choices, indicating a disconnect between knowledge and action [15]. This may be due to a lack of practical skills in applying theoretical knowledge, which could be addressed through educational programs that include practical demonstrations and peer discussions.

The mixed responses regarding dietary changes based on new information suggest that while knowledge is essential, it alone is insufficient to change behaviors. Interventions should aim to motivate and facilitate behavior change through supportive environments and accessible resources [15-17].

Study limitations

The cross-sectional nature of our study limits our ability to draw cause-and-effect conclusions between vitamin knowledge, information-seeking behaviors, and health outcomes. Longitudinal studies are needed to establish causal links and observe changes over time.

Self-reported data on vitamin knowledge and dietary behaviors may introduce biases, such as overestimating knowledge or underreporting unhealthy eating habits, potentially leading to overly positive results. In addition, our participant pool, primarily from educational institutions, might not represent the broader population of Saudi Arabia in terms of socioeconomic backgrounds and access to resources.

The online distribution of the questionnaire could exclude individuals with limited Internet or device access, potentially omitting older or lower socioeconomic groups with different vitamin knowledge and information-seeking behaviors. Conducting the study in English might also affect the accuracy of responses due to potential language barriers.

The tools and scales used to assess vitamin knowledge and information-seeking behaviors were specifically designed for this study and may lack thorough validation, potentially affecting the reliability and validity of the findings. Focusing solely on vitamins might overlook other crucial aspects of nutritional literacy, such as mineral intake, diet quality, and nutritional balance.

Study recommendations

Future research should employ a longitudinal design to better understand the dynamics of vitamin knowledge and its health impacts over time, aiming for a more diverse sample to increase the findings' applicability.

To counter self-report bias, future studies could use objective measures, such as blood tests, to validate dietary data. Expanding the research scope to encompass broader nutritional knowledge and behaviors could provide a more comprehensive view of dietary health.

There is a need to develop targeted educational programs that cater to the specific needs and preferences of different groups, using trustworthy sources and accessible through various channels, including digital platforms. Health educators and policymakers should also consider cultural and linguistic adaptations of health information to enhance understanding and engagement among non-native speakers and diverse cultural groups.

Developing and validating robust tools for measuring vitamin knowledge and information-seeking behaviors is crucial to ensure that interventions rely on accurate and reliable assessments. These tools should accommodate cultural and linguistic differences to accurately reflect health literacy in diverse populations.

## Conclusions

The results of this study highlight the challenges in enhancing vitamin knowledge and dietary habits among individuals in Saudi Arabia. By employing effective educational methods and trusted information sources, significant improvements in public health can be achieved. Our research identified substantial gaps in vitamin literacy, particularly among different age groups and those less engaged in seeking health information. Based on these findings, future research should explore new strategies to link vitamin knowledge with lasting behavioral changes in this context.

Future studies should concentrate on interactive learning, community initiatives, digital tools, and behavioral economics principles to promote healthier dietary choices among Saudis. Developing engaging educational programs, collaborating with local health experts, and implementing small incentives for healthy eating can be particularly effective. By investigating these areas, especially within Saudi communities, the aim is to cultivate a generation that is well-informed and proactive in managing their health. The ultimate goal is to foster enduring changes that lead to improved vitamin literacy and healthier lifestyles across the population.

## Figures and Tables

**Figure 1:**
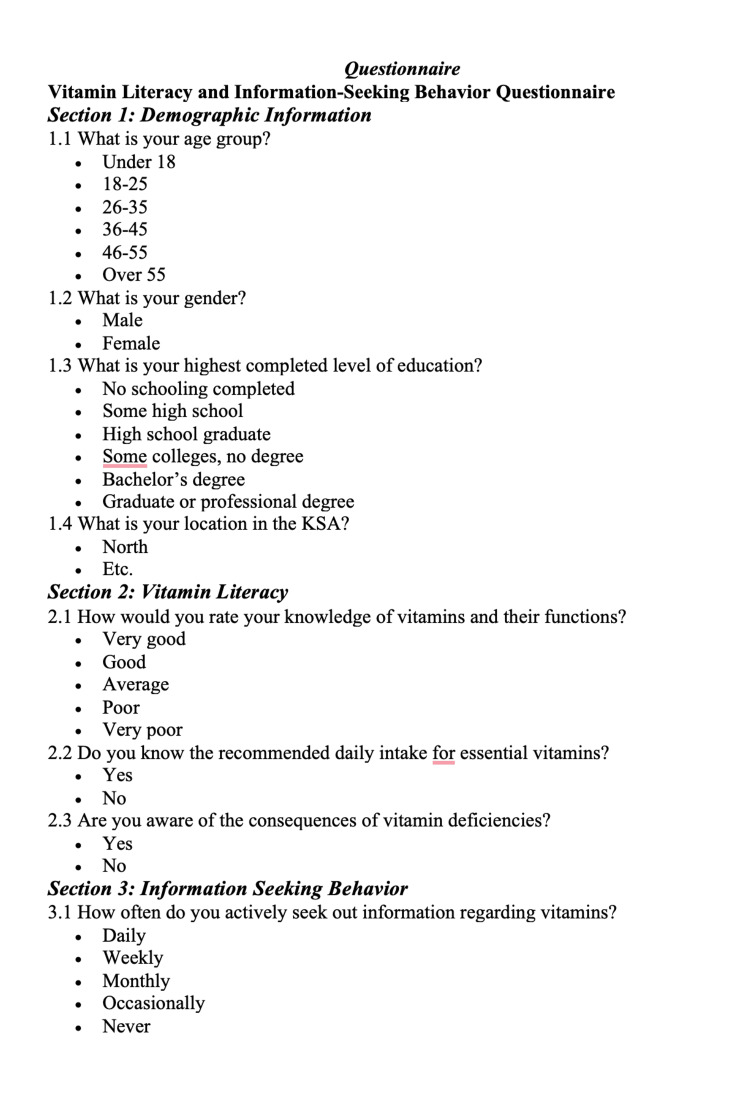
Questionnaire on Vitamin Literacy and Information-Seeking Behavior survey

**Figure 2:**
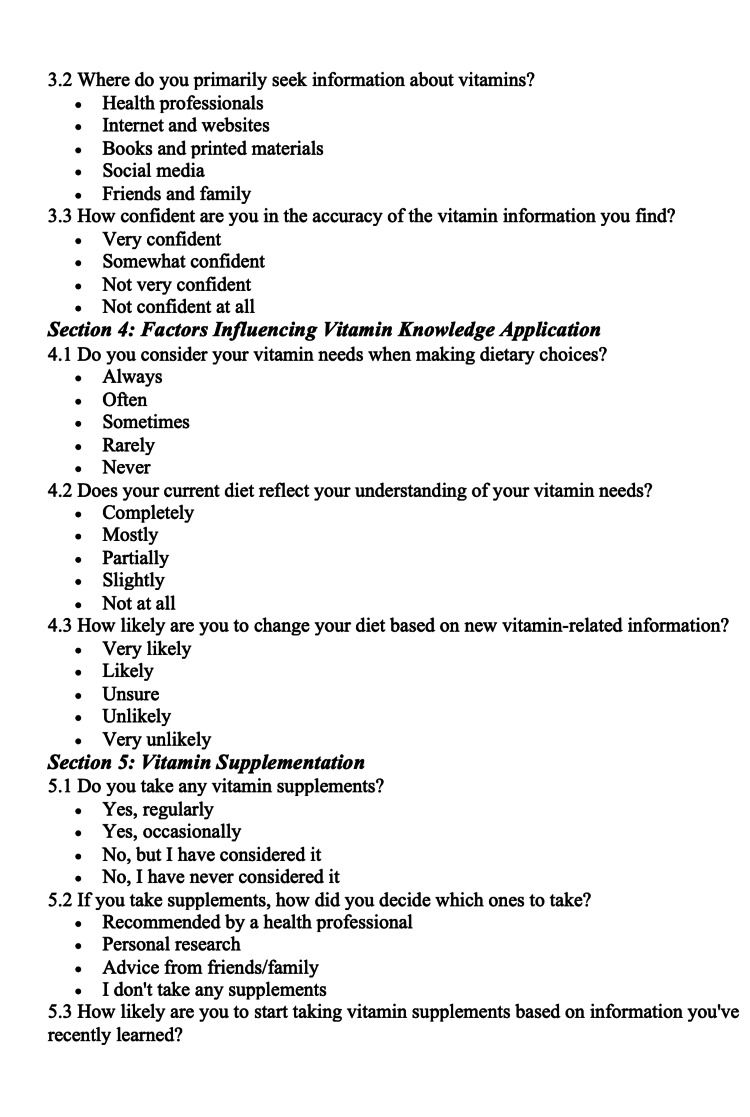
Questionnaire on Vitamin Literacy and Information-Seeking Behavior (Contd.)

**Figure 3:**
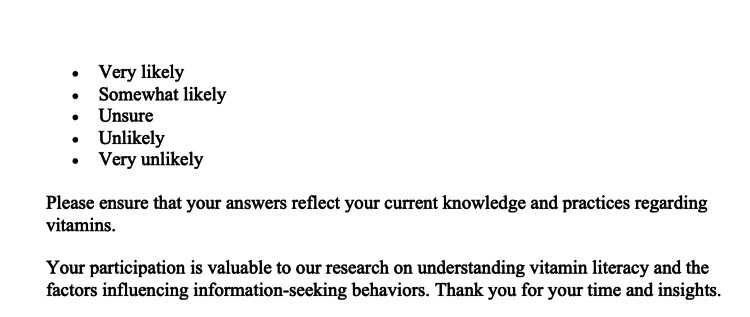
Questionnaire on Vitamin Literacy and Information-Seeking Behavior (Contd.)

## Appendices

Appendix A

Appendix B 

Appendix B 
